# Poorly differentiated thyroid carcinoma: a case report and literature review

**DOI:** 10.3389/fonc.2026.1736883

**Published:** 2026-04-01

**Authors:** Dong Zeng, Rong Wang, Zhen Liu, Junhao Hu, Huanhuan Peng, Xiaohui Wang

**Affiliations:** 1 Institute of Cancer, Sichuan Friendship Hospital, Chengdu, China; 2 Nuclear Medicine Department, Sichuan Provincial People’s Hospital, Chengdu, China; 3 Department of Hepatobiliary Surgery, General Surgery Center, General Hospital of Western Theater Command, Chengdu, China

**Keywords:** clinical symptoms, molecular characteristics, poorly differentiated thyroid carcinoma, prognosis, treatment

## Abstract

Poorly differentiated thyroid carcinoma (PDTC) is a rare and highly malignant tumor. To date, there have been very few case reports on PTCD, so its treatment methods remain controversial and require more experience. The island-like, solid-like or trabecular-like structures are manifestations of histology. Most PDTCs show positive reactions for thyroid globulin and thyroid transcription factor 1 (TTF-1) in immunohistochemical tests, and some of them also show positive reactions for p53. A 55-year-old male patient presented with a left neck mass without any obvious cause. He was diagnosed with advanced PDTC. We adopted a treatment plan combining local radiotherapy and intravenous chemotherapy. The PET/CT scan showed no increased metabolic activity throughout the body. This patient has achieved complete remission. The overall survival period is currently around one year. Additionally, we provide a comprehensive review of the disease, emphasizing incidence and definition, clinical symptoms and prognosis, molecular characteristics and treatment of PDTC, which may offer valuable insights for clinical practitioners.

## 1 Introduction

Poorly differentiated thyroid carcinoma (PDTC) is a rare and highly malignant tumor that accounts for approximately 2–15% of all thyroid cancers (1). The PDTC was initially proposed as a separate entity in 1983 and was included in the World Health Organization (WHO) classification of thyroid tumors in 2004 (2).

The incidence rates reported by PDTC vary depending on the geographical area considered, where the incidence is less than 1% of all thyroid cancers diagnosed in Japan, 2–3% in North America, and up to 15% of thyroid cancers in Northern Italy (3). Its clinical features and degree of differentiation lie between those of differentiated thyroid cancer (DTC) and anaplastic thyroid cancer (ATC) (4, 5). The median age of this disease is 59 years old, and the ratio of females to males is 1.6:1 (6). The rapid enlargement of the neck mass is the most common symptom and sign of primary thyroid cancer. In addition, symptoms such as hoarseness of voice, breathing difficulties or swallowing difficulties may also occur (7). The 5-year, 10-year, and 15-year survival rates for this disease are 50–85%, 34–50%, and 0%, respectively (8, 9).

To date, there have been very few case reports on PDTC, so its treatment methods remain controversial and require more experience. Herein, we report a case from our hospital, which will contribute to the accurate diagnosis and treatment of PDTC.

## 2 Case presentation

### 2.1 Patient

**Figure 1:**
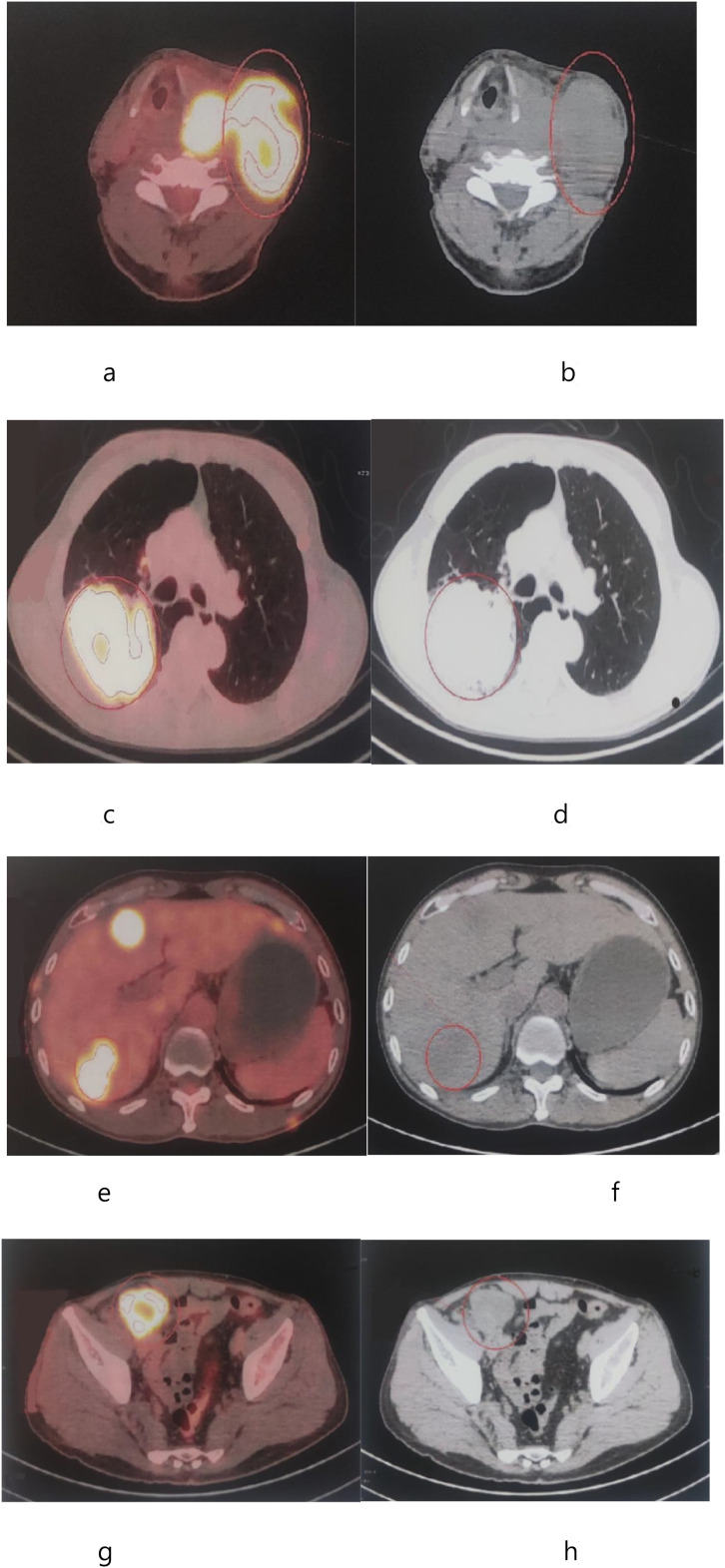
PET/CT images of the patient before treatment. **(a, b)** PET imaging shows increased [18F]-FDG uptake in the left thyroid (SUVmax 25.8) and lymph node (SUVmax 30). **(c, d)** PET imaging shows increased [18F]-FDG uptake in the right lung and pleura (SUVmax 20.6). **(e, f)** PET imaging shows increased [18F]-FDG uptake in the liver (SUVmax 30). **(g, h)** PET imaging shows increased [18F]-FDG uptake in the pelvic cavity (SUVmax 32).

A 55-year-old male patient presented with a left neck mass in January 2025 without any obvious cause. The mass was approximately 1 cm × 1 cm in size. A biopsy was performed at a local hospital, and the diagnosis was benign. Antibiotic treatment was administered, but the patient showed no significant improvement. The mass gradually increased in size. In April 2025, the patient underwent cervical lymph node ultrasound at the First Affiliated Hospital of Chongqing Medical University, which revealed an abnormal echo in the left lobe of the thyroid gland, classified as category 5, and an abnormally enlarged lymph node in the left cervical region. A thyroid biopsy was conducted, and the immunohistochemical results were CK (+), CAM5.2 (+), PAX8 (-), TTF-1 (-), TG (-), Syn (-), INSM1 (-), CD56 (+), and LCA (-). The pathological diagnosis was poorly differentiated carcinoma of the left thyroid with extensive necrosis. Genetic testing was completed for BRAF, KRAS, NRAS, BRAP, TERT, PIK3CA, and RET, all of which were wild-type. Laryngeal MRI revealed a large soft tissue mass in the left lobe of the thyroid (87x51x148 mm) that extended into the tracheoesophageal groove and encircled the left common carotid artery, suggesting the possibility of undifferentiated carcinoma. Multiple enlarged lymph nodes were found in the left neck II and III regions and the supraclavicular fossa and were considered to be metastatic lymph nodes. A PET/CT scan was conducted, revealing the following: 1. A mass in the left lobe of the thyroid gland with heterogeneous density, ill-defined margins to adjacent vessels, measuring 7.8cm x 5.9cm, and a maximum standardized uptake value (SUVmax) of 25.8. The trachea was compressed and shifted to the right. Multiple enlarged lymph nodes in the left cervical region with increased FDG metabolism suggested the possibility of a malignant tumor in the left thyroid with lymph node metastasis in the left cervical region (4.2cmx7.0cm, SUVmax 30). 2. A mass in the posterior segment of the upper lobe of the right lung, 7.9 cm × 8.9 cm, with increased PDG metabolism (SUVmax 20.6); multiple solid nodules in both lungs, some located under the pleura, the largest one in the posterior basal segment of the left lower lobe, with increased FDG metabolism; suggesting a high possibility of malignancy and multiple metastases. 3. Multiple low-density nodular masses in the liver, 4.9 cm × 3.2 cm, with increased FDG metabolism (SUVmax 30); a mass (3.9cmX4.8cm) in the right pelvic wall with increased FDG metabolism (SUVmax 32); and small nodules in the subcutaneous tissue of the left buttock, right anterior chest wall, left chest wall, and left posterior chest wall, with increased FDG metabolism, suggesting a high possibility of multiple metastases (**Figures 1A–H**).

The local hospital diagnosed the patient with advanced-stage cancer and no surgical indications. Anlotinib treatment was initiated at a dose of 12 mg once daily. The patient stopped taking the medication after only two weeks. The patient subsequently visited West China Hospital of Sichuan University and underwent a cervical lymph node biopsy. The immunohistochemical results were CK (Pan) (+), EMA (+), EGFR (+), CK7 (mainly - with a few weak +), CK5&6 (mainly + with a few weak +), p63 (-), TTF-1 (-), TG (-), Pax-8 (-), CgA (-), Syn (-), CDX2 (-), p53 (-, absent), and Ki-67 (+, >50%). *In situ* hybridization revealed EBER negativity. PD-L1 testing was completed, with results showing a TPS > 90% and a CPS > 90. The pathological diagnosis was poorly differentiated carcinoma. The patient then came to our hospital for treatment. The patient has no history of tumors in their medical records, and there is no family history of tumors. Upon admission, the patient’s vital signs were stable. After admission, tumor markers were completed, with CA199 at 7.07U/ml (normal range: 0–30 U/ml), CA125 at 109.37 U/ml (normal range: 0–35 U/ml), CEA at 1.47ng/ml (normal range: 0-4.5ng/ml), NSE at 3ng/ml (normal range:0-16.3 ng/mL),and CA242 at 3.62 U/ml (normal range: 0–20 U/ml). Liver and kidney function tests, as well as complete blood count, showed no significant abnormalities. Further thyroid function tests were performed, with results of TT3 1.78nmol/l (normal range: 1.30-3.10 nmol/l), TT4 112.0nmol/l (normal range: 66.0-181.0 nmol/l), TSH 2.07uU/ml (normal range: 0.27-4.20 uU/ml), FT3 4.90 pU/ml (normal range: 3.1-6.8pU/ml) and FT4 14.73 pU/ml (normal range: 12-22pU/ml).

### 2.2 Treatment

**Figure 2:**
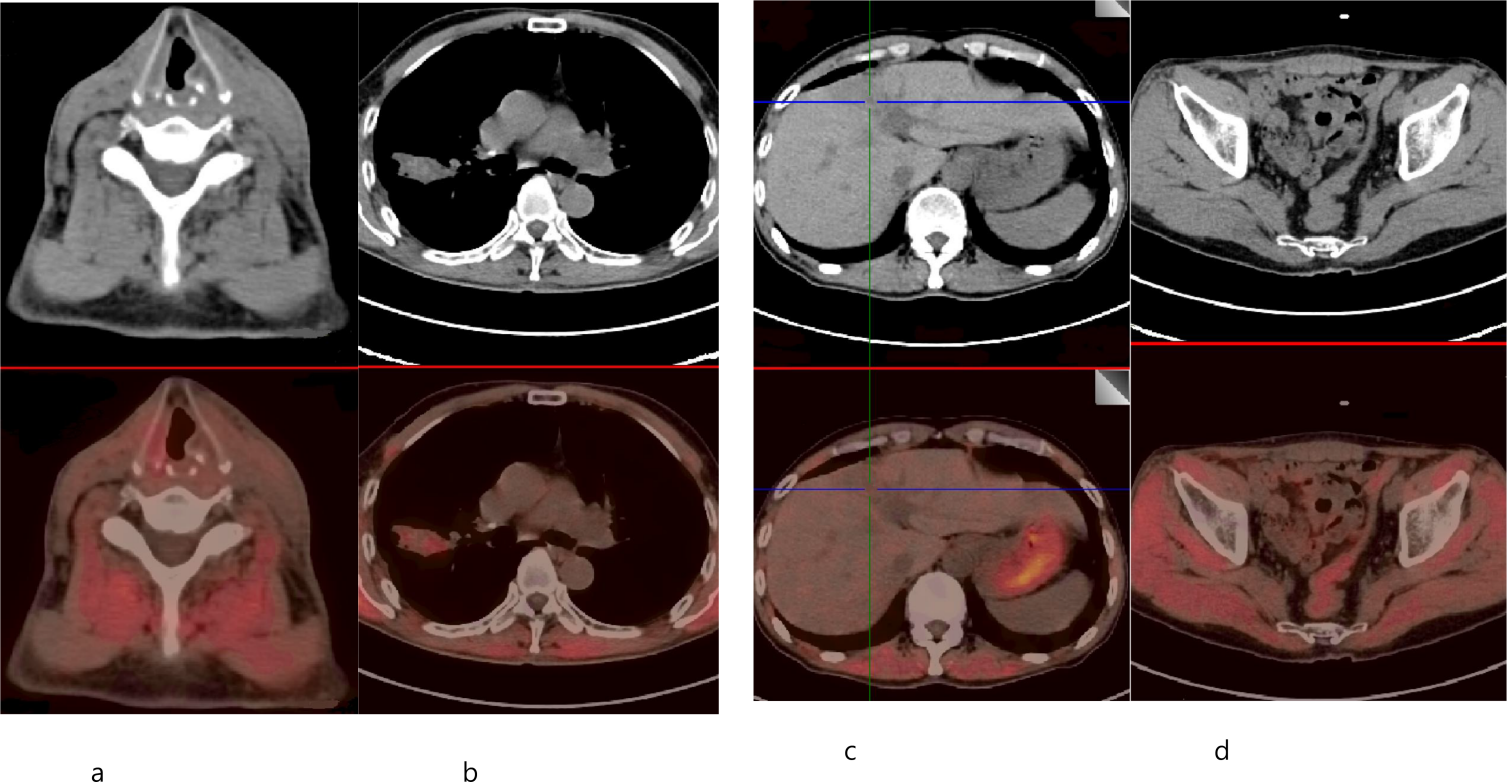
PET/CT images of the patient after treatment. **(a)** PET/CT shows that the thyroid lesion has almost completely response. **(b)** PET/CT shows that the lung lesion has almost completely response. **(c)** PET/CT shows that the liver lesion has almost completely response. **(d)** PET/CT shows that the pelvic lesion has almost completely response.

The patient has widespread systemic metastasis with extensive local tumor invasion, rendering radical resection unfeasible. Surgical evaluation indicates no indication for operative intervention. Given the patient’s condition, we adopted a treatment plan combining local radiotherapy and intravenous chemotherapy. For the thyroid and cervical lymph node metastases, stereotactic body radiotherapy (SBRT) high-dose radiotherapy was chosen first, with a total dose of 12 Gy and a fraction dose of 6 Gy, for a total of 2 sessions, which were completed every other day, followed by intensity-modulated radiotherapy (IMRT), with a total dose of 46 Gy and a fraction dose of 2 Gy, for a total of 23 sessions, once daily, five times a week. A course of the thyroid and cervical lymph node radiotherapy was completed over a period from May 9 to June 11, 2025. For the right lung lesion, SBRT was used, with a total dose of 48 Gy and a fraction dose of 8 Gy, for a total of 6 sessions, with 3 sessions per week. Lung lesion radiation therapy was administered from May 29, 2025, to June 9, 2025. For the liver lesions, SBRT radiotherapy was used, with a total dose of 48 Gy and a fraction dose of 8 Gy, for a total of 6 sessions, with 3 sessions per week. Liver lesion radiation therapy was administered from May 16, 2025, to May 28, 2025. For the pelvic lesion, SBRT radiotherapy was adopted with a total dose of 36 Gy, a fraction dose of 6 Gy, and a total of 6 sessions, with 3 sessions per week. Pelvic lesion radiation therapy was administered from May 13, 2025, to May 24, 2025. Owing to the multiple radiotherapy sites and the patient’s limited mobility in the right lower limb, we first conducted concurrent radiotherapy on the neck and pelvic lesions, followed by radiotherapy on the liver and lung lesions. Radiation therapy for the liver and pelvic lesions was administered on alternating days. During the radiotherapy period, we also administered intravenous chemotherapy, with a regimen of 440 mg of albumin-bound paclitaxel combined with 130 mg of cisplatin intravenously infused. Cisplatin treatment was completed over 3 days, with each lasting 3 weeks constituting one treatment cycle. A CT scan was conducted once during this period, and the patient’s tumor significantly shrank, with a partial response assessment. After completing radiotherapy, the patient continued with an albumin-bound paclitaxel combined with cisplatin chemotherapy regimen, completing a total of 6 cycles. The durations of the six chemotherapy sessions were as follows: from May 9th to 11th, 2025; from June 4th to 6th, 2025; from July 4th to 6th, 2025; from July 31st to August 2nd, 2025; from August 29th to August 31st, 2025; and from September 24th to 26th, 2025. The patient did not follow the three-week interval for treatment appointments consistently. In the fifth and sixth cycles, the total dose of cisplatin was reduced by 30 mg due to gastrointestinal adverse reactions. Throughout the chemotherapy period, we consistently administered pegylated recombinant human granulocyte colony-stimulating factor, and the patient did not develop bone marrow suppression. On October 22^nd^, another PET/CT scan was conducted, and the patient’s tumor was almost completely relieved (**Figure 2**). There is variable metabolic activity among the lesions: the thyroid lesion shows an SUV of 1.33, the pulmonary lesion 3.27, the hepatic lesion 1.0, and the pelvic lesion 1.5.

**Figure 3:**
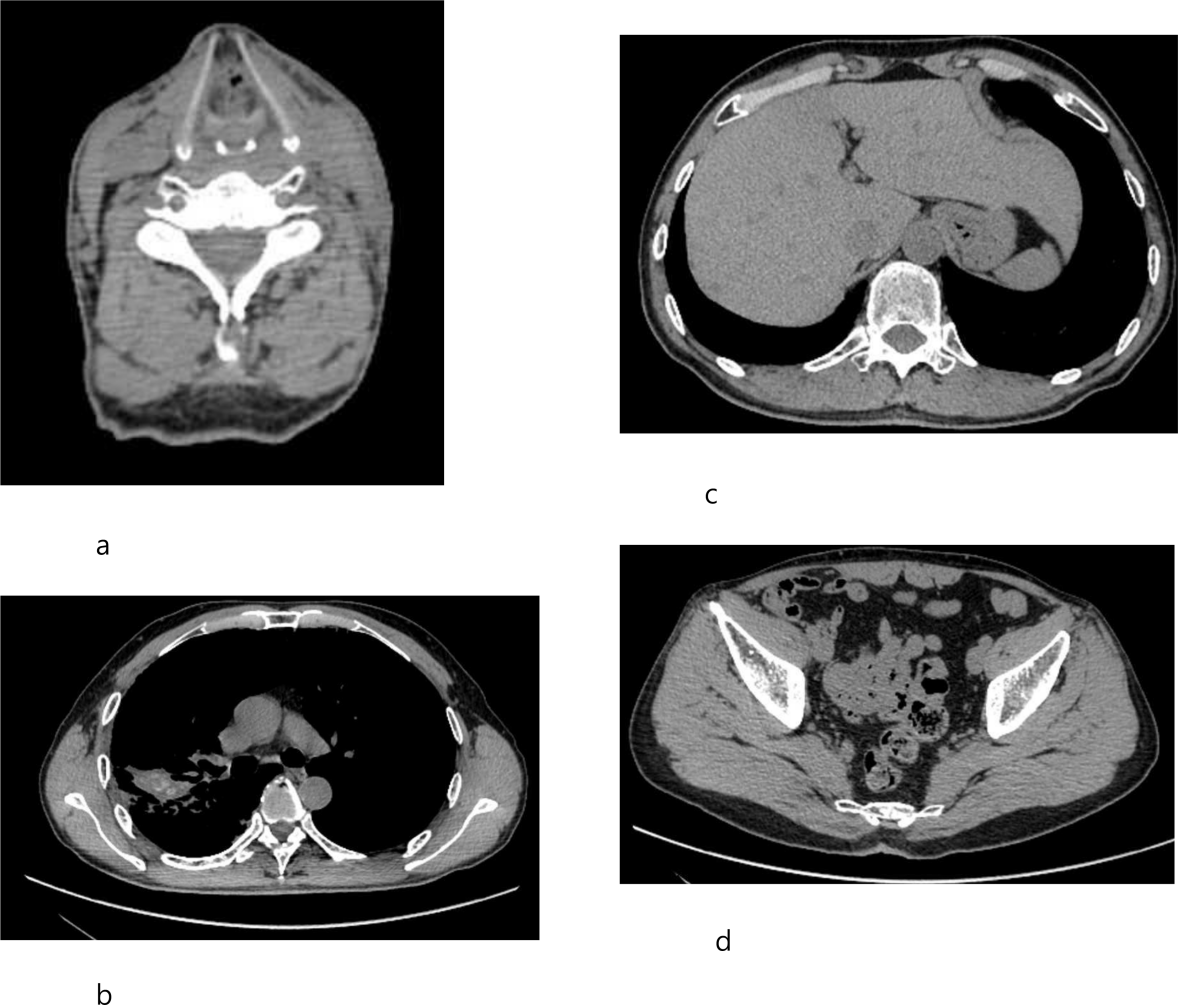
March 2026 CT follow-up scan. **(a)** The original thyroid lesion shows no progression. **(b)** The original lung lesion shows no progression. **(c)** The original liver lesion shows no progression. **(d)** The original pelvic lesion shows no progression.

On March 4, 2026, the patient returned to the hospital for a follow-up examination. No recurrence was found in the previous lesion sites of the thyroid gland, right lung, liver or pelvic region. (**Figures 3A–D**).

**Figure 4:**
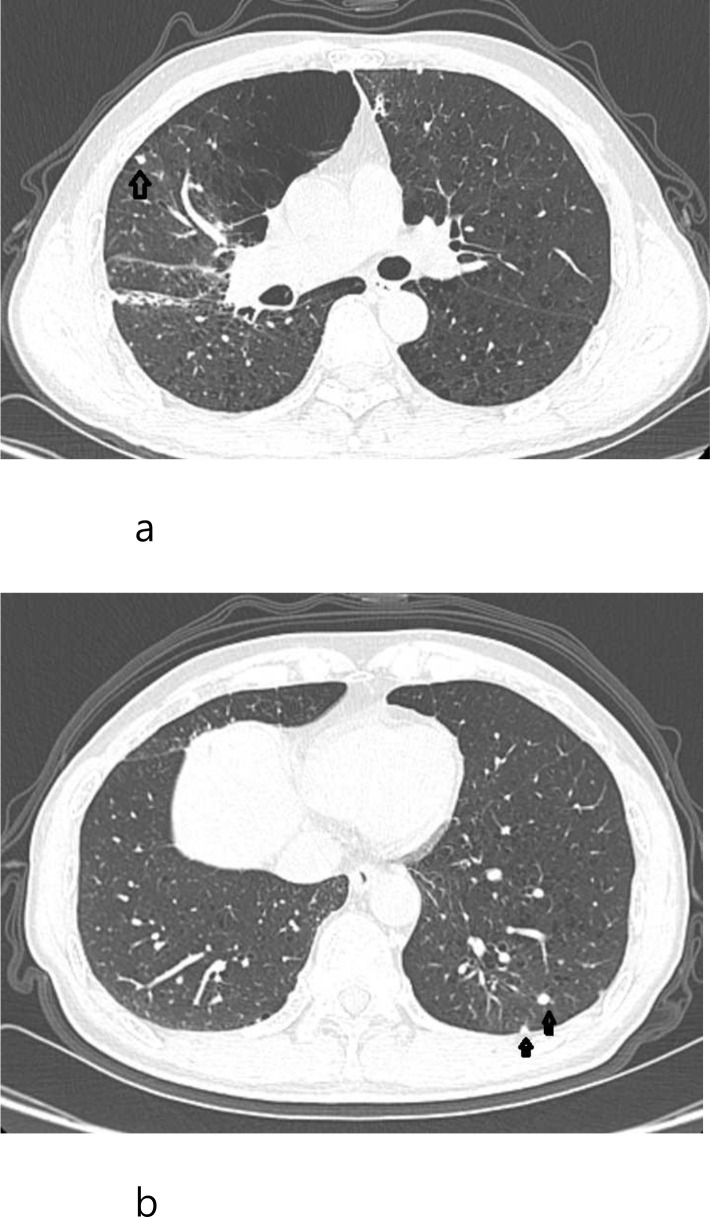
March 2026 CT follow-up scan of lung. **(a)** The original thyroid lesion shows no progression. **(a)** A nodule is visible in the right lung at the arrow. **(b)** Two nodules are visible in the left lung at the arrow.

However, several small nodules were found in the upper lobe of the right lung and the lower lobe of the left lung (**Figures 4A, B**).

**Figure 5:**
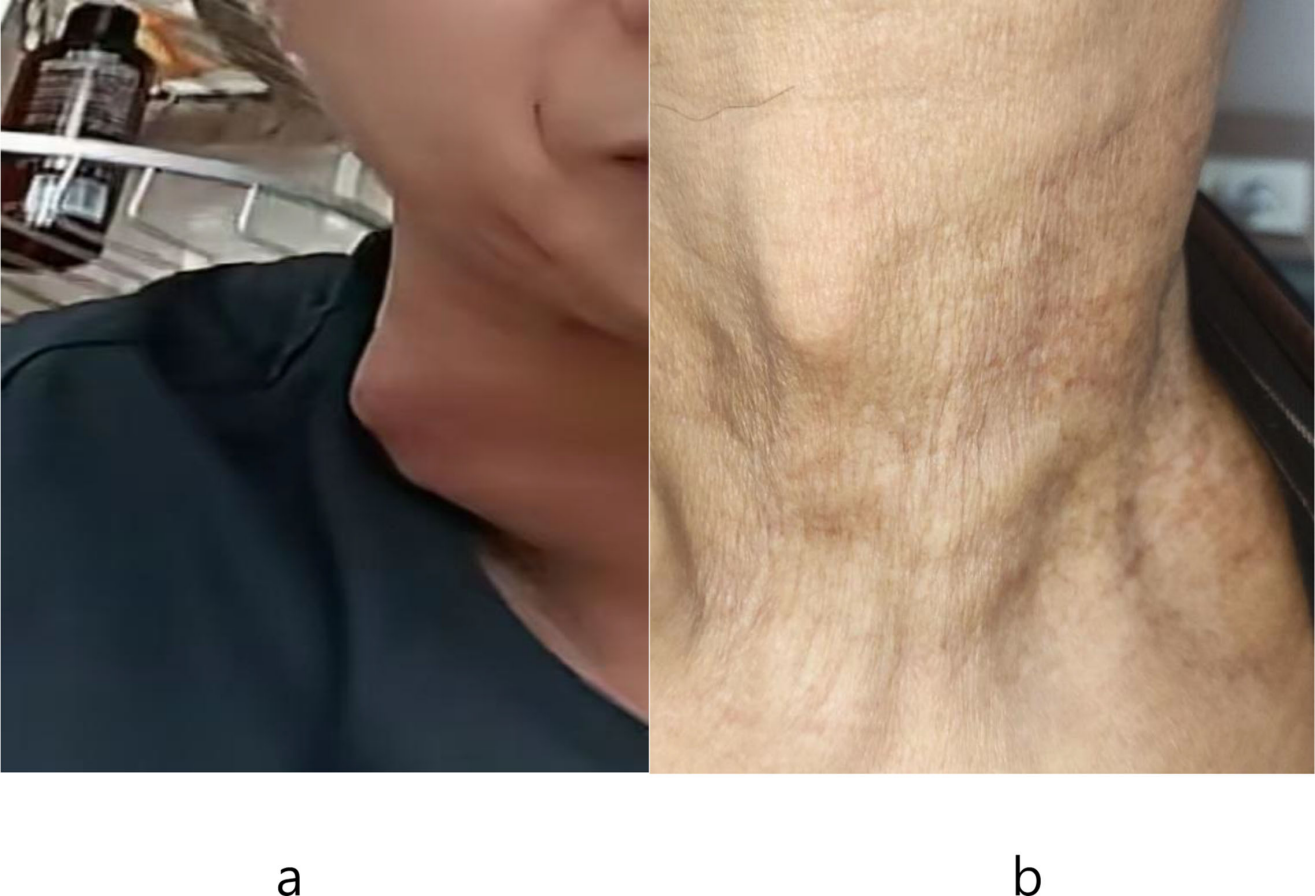
The appearance of the patient’s neck lesion. **(a)** The patient’s self-taken photo before treatment. **(b)** Neck appearance in March 2026.

Based on the patient’s history, these are considered to be tumor metastases. Further analysis of the reasons: The patient self-discontinued capecitabine after only 3 months of oral administration following intravenous chemotherapy. Since the initial diagnosis of advanced-stage tumor, the patient has survived for nearly one year with a high quality of life and is deeply grateful for our treatment (**Figures 5A, B**).

## 3 Discussion

### 3.1 Incidence and definition

PDTC is an individual form of thyroid carcinoma derived from follicular epithelial cells and is believed to be relatively rare (10). The incidence of PDTC is only 2–15% of all thyroid cancers, and the incidence rate varies across different regions (11). PDTC ranks between DTC and ATC with respect to the degree of differentiation. PDTC was defined on the basis of the mitotic index and the presence of necrosis in a tumor with pathological or immunohistochemical evidence of follicular differentiation by the Memorial Sloan Kettering Cancer Center (MSKCC) in 2006 (12). A group of pathologists defined the PDTC diagnostic indicators as follows: 1) solid/trabecular/insular pattern of growth, 2) absence of conventional nuclear features of papillary carcinoma, and 3) presence of at least one of the following features: mitotic activity ≥3/10 high-power microscopic fields (HPFs), tumor necrosis and convoluted nuclei (13). These criteria were adopted as a uniform definition for histopathological diagnosis in the WHO Classification of Endocrine Tumors in 2017 (14). The histopathological examination of hematoxylin-eosin sections plays the most crucial role in the diagnosis of PDTC, while immunohistochemistry seems to have a relatively minor role in the classification of this tumor (15). Immunohistochemical techniques have long been regarded as capable of increasing the accuracy of diagnosis by narrowing the range of differential diagnoses and reflecting the progression of the disease. However, specific immunohistochemical markers for detecting PDTC have not yet been established (16).

### 3.2 Clinical symptoms and prognosis

A rapidly enlarging neck mass is the most common symptom and sign of primary thyroid cancer. In addition, symptoms such as hoarseness of voice, breathing difficulties or swallowing difficulties may also occur. However, these clinical manifestations are not specific. In the early stages of the disease, they may be misdiagnosed as benign tumors, similar to this patient’s case. The majority of patients are elderly, and a slight female preponderance is observed (17). The vast majority of patients present with lymph node metastasis at initial diagnosis, and more than one-third of patients have distant metastasis (6). Like our patient in this case, distant metastasis to the lungs, liver, and pelvis occurred at the initial treatment. Currently, the eighth edition of the American Joint Committee on Cancer and UICC staging system is primarily used, which provides more accurate predictions of overall survival than the seventh edition does (18). The 5-year, 10-year, and 15-year survival rates for PDTC patients are approximately 50% - 85%, 34% - 50%, and 0%, respectively (19). In the multivariate analysis, extrathyroidal extension was the only parameter that had a significant impact on the overall survival rate (9). However, some multivariate analyses indicated that age, Ki-67 index, invasion depth, lymph node metastasis, distant metastasis, and the ratio of platelets to lymphocytes were independent risk factors (20).

### 3.3 Molecular characteristics

Currently, second-generation sequencing technology is being utilized to explore the molecular characteristics of PDTC. The mutation burden significantly increases from PTC to PDTC (21). In PDTC, the most common driver gene mutations are mutually exclusive mutations in BRAF and RAS. RAS activates the MAP kinase pathway as well as the PI3K/AKT pathway. BRAF mutations are present in 15% to 27% of PDTC patients and are associated with specific activation of the MAP kinase pathway (22, 23). RAS and BRAF point mutations are associated with a worse prognosis and an increased rate of distant and regional lymph nodes metastases (15). As another common alteration in PDTC, the occurrence rate of TERT promoter mutations is 40%. Mutations in the TERT promoter gene in PDTC usually indicate that the tumor has aggressive characteristics and predict a poor prognosis (21). In PDTC, the most common type of tumor suppressor gene mutation is TP53, followed by ATM. Other mutations, such as AEIF1AX, ABL1, GNAS, HNF1A, RECQL4, IDH1, STK11 and TSHR, can also be observed (24). Fourteen percent of PDTC cases exhibit chromosomal rearrangements, such as RET/PTC, PAX8/PPARγ and fusions of ALK (21). Programmed cell death ligand 1 (PDL1) plays a vital role in immune escape (25, 26). In a small-scale study, PDTC typically shows negative PD-L1 expression, but this patient’s PD-L1 expression tumor proportion score (TPS) was greater than 90% (27).

### 3.4 Treatment

Among traditional treatment methods, surgery remains the primary treatment approach. Total thyroidectomy combined with lymph node dissection is the preferred treatment method for PDTC (28). However, more than 50% of patients with PDTC have already developed extensive cervical diseases at the time of diagnosis. Adjuvant therapy is very important. Some studies suggest that radioactive iodine (rai) is an effective treatment measure for postoperative conditions (29). Owing to the lack of effective treatment methods, rai has been used for the majority of PDTC patients with distant metastasis (28). However, some researchers have failed to demonstrate that RAI therapy offers any overall survival benefits to patients with PDTC. The presence of poorly differentiated areas significantly reduces the retention of radioactive substances and therapeutic efficacy (30). External beam radiation therapy (EBRT) is recommended for postoperative adjuvant treatment of PDTC, such as (1) tumors >4 cm with minimal extrathyroidal extension without distant metastasis, (2) extensive extrathyroidal extension of any tumor size, and (3) regional lymph node metastasis (31). SBRT can deliver high-dose radiation with submillimeter precision within a limited number of fractions, thereby minimizing radiation exposure to surrounding healthy tissues to the greatest extent possible (32). Studies have reported that the combination of SBRT and PDL1 inhibitors for thyroid cancer has a significant antitumor effect (33). IMRT can ensure that the high-dose area closely conforms to the complex geometric shape of the target; it can create a steep dose gradient between the target area and nearby critical structures; and without sacrificing the coverage required for the target area, it can relatively reduce the clinically significant dose received by the normal tissues in the surrounding nontarget areas (34). IMRT has also been used to treat PDTC, but research data are sparse (30). Chemotherapy was once thought to have limited effectiveness in patients with thyroid cancer. However, some studies have shown that the combination of liposomal doxorubicin and cisplatin can achieve significant tumor shrinkage (35). The patient achieved 10 months of complete remission with the doxorubicin–cisplatin combination, followed by 5 months of control with the paclitaxel–carboplatin regimen (36). Therefore, taxanes are also employed as part of the therapeutic regimen for PDTC. We adopted a combined approach of SBRT and IMRT, integrating their respective advantages while simultaneously incorporating chemotherapy, ultimately achieving remarkable results. Currently, molecular targeted therapy is widely used in cancer treatment. Research on targeted therapy for thyroid cancer has focused mostly on tyrosine kinase inhibitors. Lenvatinib is a multitarget tyrosine kinase inhibitor. A Portuguese research team used lenvatinib to treat PDTC and achieved 100% disease control, with partial response and stable disease accounting for 12.5% ​​and 87.5%, respectively (37). A PDTC patient who developed meningeal metastasis after multiple lines of treatment also achieved long-term disease control with lenvatinib (38). Its clinical applications sometimes include hypertension, diarrhea, fatigue, and especially proteinuria (39). According to a single institution retrospective analysis, cabozantinib is also an effective and reasonably well-tolerated treatment option for PDTC (40). The European Medicines Agency has approved sorafenib as a therapy (41). The combination of the BRAF inhibitor dabrafenib and the Mek inhibitor trametinib has been approved by the U.S. Food and Drug Administration for the treatment of anaplastic thyroid cancer with the BRAF-V600E mutation (42). For RET fusion or NTRK rearrangement, pralsetinib and larotrectinib, respectively, demonstrated antitumor efficacy (43, 44). Currently, immunotherapy is advancing rapidly. The overexpression of PD-L1 in PDTC cells has been confirmed, and the combination of lenvatinib and pembrolizumab is an effective treatment (45, 46). The patient highly expressed PDL1, but he was worried about adverse reactions to immunotherapy, so he did not choose immunotherapy. Viral therapy is a novel cancer treatment method that may benefit patients in the future (47).

## 4 Conclusion

We present a case of PDTC with multiple metastases throughout the body. After radiotherapy combined with chemotherapy, an excellent antitumor effect was achieved. Additionally, we provide a comprehensive review of the disease, aiming to offer valuable insights for clinical practitioners.

## Data Availability

The original contributions presented in the study are included in the article/supplementary material. Further inquiries can be directed to the corresponding author.
